# Performance Analysis of Blended Membranes of Cellulose Acetate with Variable Degree of Acetylation for CO_2_/CH_4_ Separation

**DOI:** 10.3390/membranes11040245

**Published:** 2021-03-29

**Authors:** Ayesha Raza, Sarah Farrukh, Arshad Hussain, Imranullah Khan, Mohd Hafiz Dzarfan Othman, Muhammad Ahsan

**Affiliations:** 1 Department of Chemical Engineering, School of Chemical and Materials Engineering, National University of Sciences and Technology, Islamabad 44000, Pakistan; Sarah.farrukh@scme.nust.edu.pk (S.F.); Arshad.hussain@scme.nust.edu.pk (A.H.); Imranullah.khan@scme.nust.edu.pk (I.K.); ahsan@scme.nust.edu.pk (M.A.); 2 Advanced Membrane Technology Research Centre (AMTEC), Faculty of Chemical and Energy Engineering, University of Technology Malaysia, Skudai 81310, Malaysia; hafiz@petroleum.utm.my

**Keywords:** global warming, natural gas, blended membranes, CTA, CDA

## Abstract

The separation and capture of CO_2_ have become an urgent and important agenda because of the CO_2_-induced global warming and the requirement of industrial products. Membrane-based technologies have proven to be a promising alternative for CO_2_ separations. To make the gas-separation membrane process more competitive, productive membrane with high gas permeability and high selectivity is crucial. Herein, we developed new cellulose triacetate (CTA) and cellulose diacetate (CDA) blended membranes for CO_2_ separations. The CTA and CDA blends were chosen because they have similar chemical structures, good separation performance, and its economical and green nature. The best position in Robeson’s upper bound curve at 5 bar was obtained with the membrane containing 80 wt.% CTA and 20 wt.% CDA, which shows the CO_2_ permeability of 17.32 barrer and CO_2_/CH_4_ selectivity of 18.55. The membrane exhibits 98% enhancement in CO_2_/CH_4_ selectivity compared to neat membrane with only a slight reduction in CO_2_ permeability. The optimal membrane displays a plasticization pressure of 10.48 bar. The newly developed blended membranes show great potential for CO_2_ separations in the natural gas industry.

## 1. Introduction

Natural gas has progressively replaced fossil fuels as the green energy source for modern power plants [1,2]. However, depending on the geological location, raw natural gas varies significantly in composition and may contain 10–40 mole% CO_2_ [3,4]. The separation of CO_2_ from natural gas is not only essential to lessen the concentration of CO_2_ emission in the atmosphere but also to enhance the calorific value of the fuel, to decrease the pipeline corrosion, and to reduce the volume of gas which is to be transported through pipelines. The environmental and economic benefits of membrane technology are the foremost reasons of its tremendous progress in the last few decades compared to the other conventional separation techniques, such as amine absorption [5].

Cellulose is the biodegradable natural polymer mainly obtained from wood and cotton [6]. The glucoside repeat units of cellulose contain 3 hydroxyl groups that are responsible for the strong intermolecular hydrogen bonding. The reaction of cellulose with acetic anhydride and acetic acid in the presence of catalyst (H_2_SO_4_) produces a new class of materials known as cellulose acetates. Cellulose acetate (CA) has been used for membrane preparation from the beginning. In the late 1950s, Loeb and Sourirajan patented the development of the asymmetric CA osmotic membranes for sea water desalination [7]. Well ahead in 1970s, CA membranes were adapted for gas separation, mainly for CO_2_ removal from natural gas and hydrogen purification [8]. Few years later in the mid-1980s, the first commercial CA membrane process was developed for CO_2_ removal from natural gas, and since then it is dominating the market of CO_2_ membrane separation [9]. The success of CAs is linked to its easy availability, low cost, and stability (both mechanical and chemical). Currently, it is the most widely used commercial polymer for CO_2_ separation. In 2012, CA accounted for up to 80% of the total membrane technology market for natural gas processing [10]. Currently, two leading companies, i.e., UOP Separex and Cynara, are providing CA membranes for natural gas separation [11].

Three different types of cellulose acetates with variable degree of acetylation, namely cellulose monoacetate (CMA), cellulose diacetate (CDA), and cellulose triacetate (CTA), can be produced based upon the number of hydroxyl groups of cellulose repeat unit replaced by acetyl groups. The degree of acetylation ranges from 1 to 3, which is the average number of acetyl groups present per repeat unit of the polymer [12,13]. Although CMA, CDA, and CTA have similar structures, their crystallinity as well as thermal and mechanical properties are quite different. Moreover, the gas transport properties of the CA membranes are very sensitive to the degree of acetylation. Puleo et al. reported that CA membranes with a higher degree of acetylation are more CO_2_ permeable but less CO_2_/CH_4_ selective [13].

Several research papers were found regarding the fabrication and investigation of gas transport properties of different CAs membranes (CMA, CDA, and CTA) [13,14,15,16,17,18,19,20,21,22,23,24]. Houde et al. studied the effect of exposing the CDA membrane to high pressure CO_2_ prior to the permeability measurements. They found that the gas separation performance of the membrane was dependent on exposure time as well as exposure pressure. Their best conditioned membrane, which was exposed to CO_2_ at 27 atm for 5 days, showed CO_2_ permeability of 4.57 barrer with 25.4 CO_2_/CH_4_ selectivity at 10 atm and 35 °C [17]. Mubashir et al. fabricated CA mixed matrix membrane (MMM) by incorporating NH2-MIL-53(Al) in CA matrix. MMM loaded with 15 wt.% loading exhibited excellent CO_2_ permeability of 52.6 barrer with 28.7 CO_2_/CH_4_ selectivity [21]. In another work, Kim and coworkers incorporated nanoporous silicate flakes in the polymer matrix of CA. They found significant improvement in the gas permeability without notable change in CO_2_/CH_4_ selectivity. This might be due to the creating of a highly tortuous path for slower molecules [22]. 

Recently, two important publications may reignite the interests in CA membranes for CO_2_ separation. One of them was from Koros and his coworkers who investigated the plasticization phenomena of a commercial CTA membrane provided by Cameron, a Schlumberger Company for natural gas sweetening [23]. The membrane not only possessed an attractive CO_2_ permeance and CO_2_/CH_4_ selectivity but also exhibited high tolerance against aromatic contents. The other was from Lin and his team who investigated the effect of CDA film thickness and crystallization on the CO_2_/CH_4_ separation properties. Thin films of CDA with variable thicknesses were prepared. They found that the reduction in film thickness from 20 um to submicron inhibits a crystallinity from 0.34 to 0.02, which resulted in a 130% enhancement in the gas permeability while retaining the CO_2_/CH_4_ selectivity [24].

Among different polyimides, CA has been extensively used in the market of gases separation processes. However, CA membrane exhibits relatively good selectivity values but lower gas permeability. To make the membrane more productive, one has to transform the membrane materials. To our best knowledge, this would be the first time that CA membranes are transformed by blending CAs of variable degrees of acetylation. We hypothesize that the blend of CDA with CTA will improve the performance because both the polymers have same chemical structure but different crystallinity and density. Moreover, the backbone chains of both the polymers are same, which eliminate the issues of compatibility and miscibility. The aim of this work is to investigate how the transport properties will be affected when the backbone chain of CTA, which contains bulky pendant groups (acetyl), interacts with the CDA, which has the same backbone composition but smaller pendant groups (hydroxyl). A permeation study is carried out as a function of blend composition. At the end, separation performances of all the fabricated membranes are compared based on Robeson’s upper bound curve. The plasticization pressure is recorded for the optimized sample.

## 2. Materials and Methods

### 2.1. Materials Used

Cellulose triacetate (CTA, degree of substitution ≈ 2.84) and cellulose diacetate (CDA, degree of substitution ≈ 2.45) were bought from Selectophore Merck, Malaysia. N-methyl-pyrrolidone (NMP) was purchased from Sigma Aldrich, Malaysia. 

**Figure 1:**
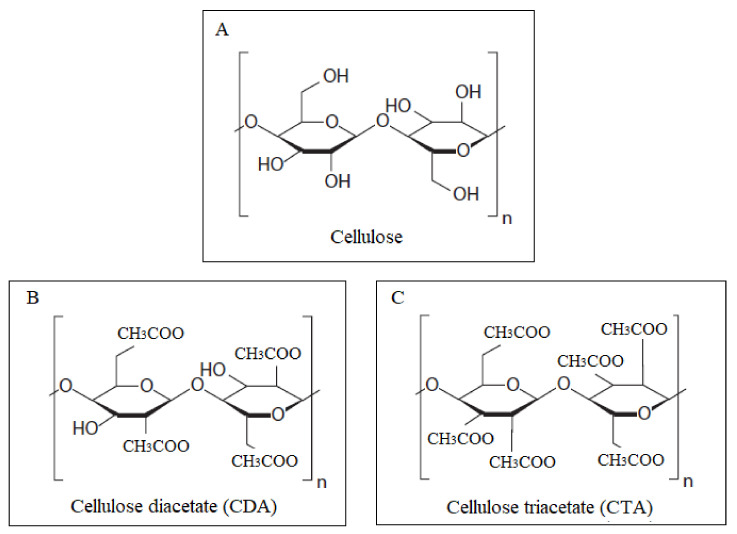
Molecular structures of (**A**) cellulose, (**B**) cellulose diacetate (CDA), and (**C**) cellulose triacetate (CTA).

Figure 1 shows the chemical structures of different Cas, and Table 1 tabulates their crystallinity and their mechanical and thermal properties [13].

### 2.2. Membrane Fabrication

Solution mixing and solution casting techniques were used for membrane preparation. Firstly, 80 wt.% of CTA and 20 wt.% of CDA were added in an appropriate amount of NMP to prepare a 10 w/v solution. Afterwards, the polymer solution was stirred for 12 h, followed by degassing overnight at room temperature. The degassed polymer solution was then casted on a clean glass slab, and it then underwent heat treatment at 120 °C for 24 h. After drying, membrane was peeled off from the glass slab labeled as CTA:CDA (80:20). Similar method was followed to prepare CTA:CDA (100: 0), CTA:CDA (60:40), CTA:CDA (50:50), and CTA:CDA (0:100). The resultant membranes were then used for characterization and gas permeation testing.

### 2.3. Membranes Characterization

Fourier-transform infrared spectroscopy (FTIR, spectrum 100) was used to determine the functional groups and type of interaction between CTA and CDA polymeric chains. The analysis was carried out in the wave number range of 4000–600 cm^−1^. The crystallinity of fabricated membranes was determined through X-ray diffraction (XRD, AG-XEUS). For the XRD analysis, membranes were scanned from a 2-theta value of 5–40°. A universal testing machine (UTM, AG-XPlus Shimadzu) was used to examine the tensile strength and flexibility of the fabricated samples. Rectangular strips of fabricated samples were mechanically tested according to ASTM-D882-02 at a strain rate of 0.5 mm/min. The morphology of fabricated membranes was verified by a scanning electron microscope (SEM, ZEOL-JSM-6490A) analysis at high resolutions.

### 2.4. Gas Permeation Study

A permeation study of the fabricated membranes was carried out using single gas. A bubble flow meter (Agilent, ADM1000, Santa Clara, CA, USA) was used to record the permeation rate of each gas. All the measurements were carried out at room temperature (25 ± 1 °C) and a transmembrane pressure difference of 5 bar (72.52 psi ± 0.3). Four samples of each membrane type were tested, and the average value was reported in this work. 

Gas permeability was calculated by following equation:

(1) $$P_{i}=\;\frac{Q_{i}\;L}{A△P}$$

where *P_i_* is the gas permeability in Barrer (1 barrer = 1 × 10^−10^ cm^3^ (STP)·cm/cm^2^·s·cm Hg); *i* represents the penetrating gas; the volumetric flow rate of the permeated gas molecules is signified by *Q_i_* [cm^3^ (STP)/s]; *L* is the membrane thickness (cm); *A* is the effective area of the membrane (cm^2^); and the pressure difference (cm Hg) across the membrane is represented by △*P*.

CO_2_/CH_4_ selectivity was calculated by taking the quotient of permeability of both separating gases as shown in Equation (2):

(2) $$\alpha (\frac{{\mathrm{CO}}_{2}}{{\mathrm{CH}}_{4}})=\;\left(P_{\mathrm{CO}2}\right)/\left(P_{\mathrm{CH}4}\right)$$

where *P*_CO2_ and *P*_CH4_ are the CO_2_ and CH_4_ permeability respectively, and *α* represents membrane selectivity.

## 3. Results and Discussions

### 3.1. Membrane Characterization

#### 3.1.1. FTIR Analysis

**Figure 2:**
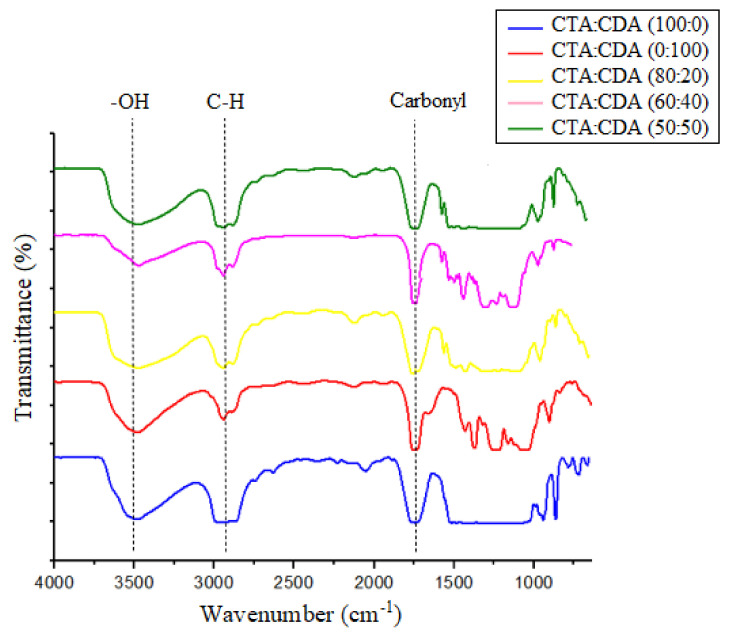
FTIR spectrum of fabricated membranes.

The FTIR analysis of fabricated membranes is illustrated in Figure 2. Major peaks of pristine and blended membranes correspond to the O-H, C-H stretching, and C=O functional groups observed at the wave numbers of 3482.88 cm^−1^, 2925.03 cm^−1^, and 1725.81 cm^−1^ respectively [25,26]. No additional peak was observed in the CTA/CDA blended membrane compared to the pristine membranes, which justifies the physical interaction between the chains of CDA and CTA.

#### 3.1.2. XRD Analysis

**Figure 3:**
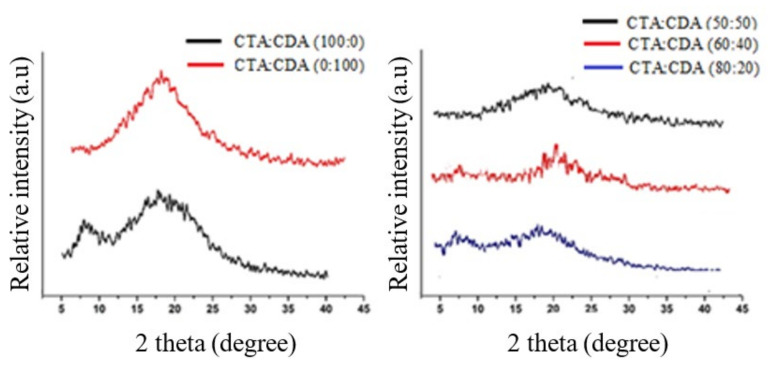
XRD analysis of fabricated membranes.

Figure 3 shows the XRD pattern of fabricated membranes. The presence of broad diffraction peaks in the XRD pattern of CTA as well as CDA confirmed their semicrystalline nature. CTA displayed two major peaks at 2θ values of 7° and 18°, which characterize the crystalline and amorphous regions, respectively [26,27,28]. However, the peak corresponding to the crystalline region (2θ = 7°) is not very prominent in CDA, which verifies that CTA is much more crystalline than CDA. These results are in good agreement with the literature [13,24,28].

Moreover, the XRD pattern of blended membranes reveals that the peak corresponding to the crystalline region (2θ = 7°) gradually disappeared by increasing the percentage of CDA in a blend from 20 to 50%. These results confirm the decrement in the crystallinity with the rise of CDA concentration in the blend. 

#### 3.1.3. SEM Analysis

**Figure 4:**
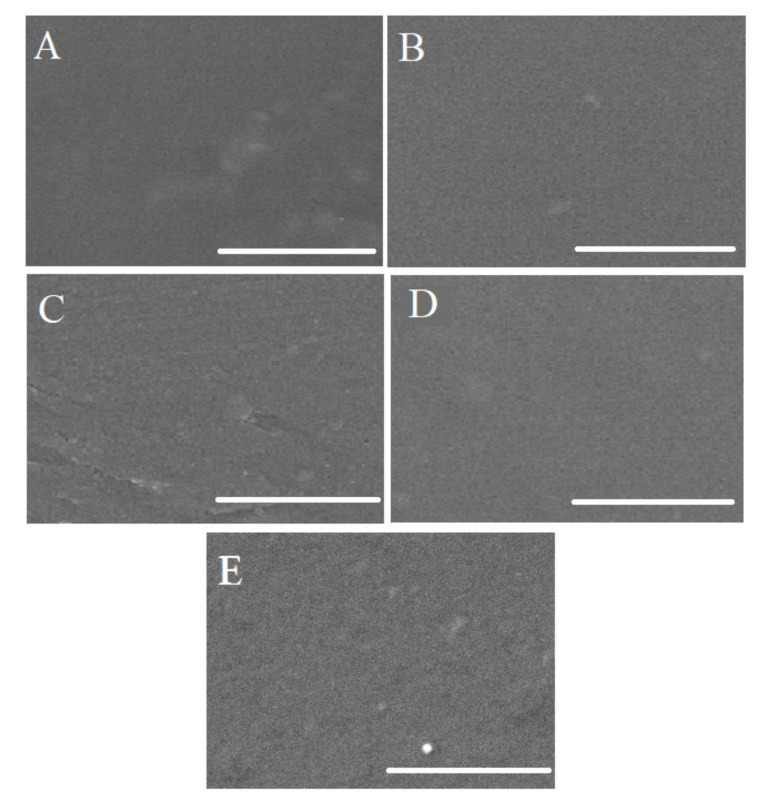
Surface morphology of (**A**) CTA:CDA (100:0), (**B**) CTA:CDA (0:100), (**C**) CTA:CDA (80:20), (**D**) CTA:CDA (60:40), and (**E**) CTA:CDA (50:50).

**Figure 5:**
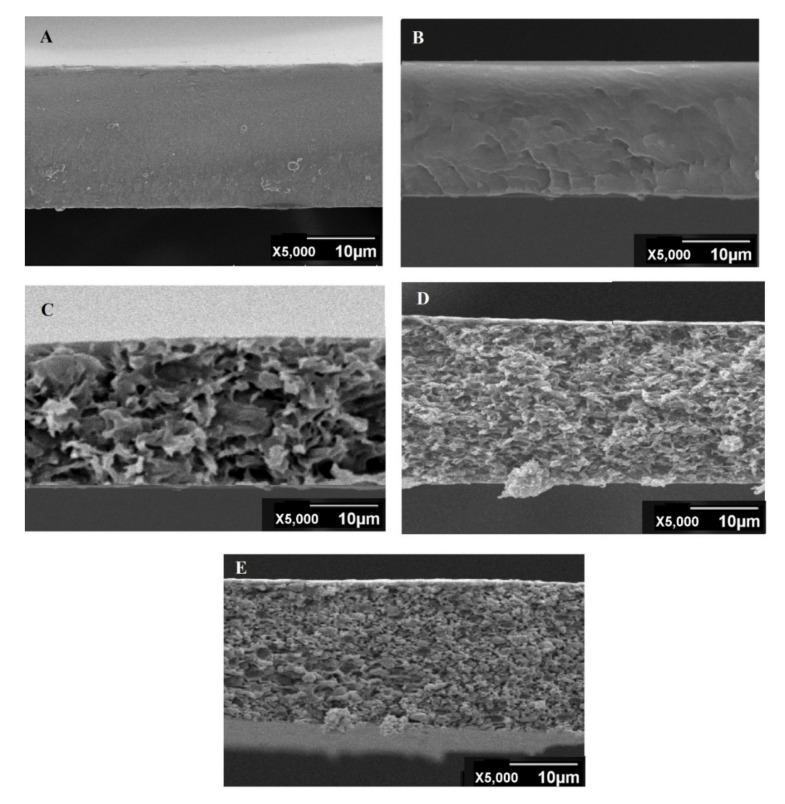
Cross-section SEM images of (**A**) CTA:CDA (100:0), (**B**) CTA:CDA (0:100), (**C**) CTA:CDA (80:20), (**D**) CTA:CDA (60:40) and (**E**) CTA:CDA (50:50).

Figure 4 and Figure 5 display the surface and cross-section morphologies of all fabricated membranes. Surfaces of all the membranes were dense and defect free. Formation of the homogenous blend and a good interfacial interaction were confirmed from the SEM images. Referring to the SEM results, the pristine CTA and CDA membranes depicted a closely packed, dense, and symmetric structure. In the case of CDA, the results are due to the presence of strong hydrogen bonding between polymer chains, which may give the foundation of closely packed and completely dense structure. In the latter case, highly polar and large-sized acetate pendant groups of CTA may anchor the nearby molecules and permit the polymer chains to develop strong secondary forces, resulting in the formation of closely packed structure [26].

Cross-section morphologies of all the blended membranes displayed an asymmetric loosely packed structure in between dense skin layers (Figure 5). Improvement in the packing density of polymeric chains and reduction in the free volume was observed by decreasing the percentage of CTA in blended membranes. Minimum voids were observed in CTA/CDA (50:50). Generally, steric hindrance and van der Wall forces between chains are the two major factors that contribute towards the packing density. Steric hindrance is a disability that leads to the formation of loosely packed structure, whereas van der Wall forces lead towards better packing. These results are attributed to the steric hindrance, which is considered as a dominant factor in the CTA/CDA blended membranes. The greater the number of bulky groups (such as acetate), the greater the steric hindrance, and the more voids there are in the cross section of the polymer [26].

#### 3.1.4. Mechanical Properties

The mechanical performance of fabricated membranes is illustrated in Table 2. The tensile strengths of CDA and CTA were found to be at 32.89 ± 0.41 and 38.55 ± 0.22 MPa, respectively. However, CDA is more flexible compared to CTA. The results clearly indicate that the mechanical properties of fabricated membranes are very sensitive to the degree of acetylation. This might be because of the higher crystallinity of CTA compared to CDA [28,29,30]. Generally, the crystalline portion imparts strength to the semicrystalline polymer, whereas the amorphous region is mainly responsible for flexibility. The same trend was observed by Puleo et al. [13]. They reported that a higher degree of acetylation yielded stiffer and stronger chains.

Consistent with the aforementioned analyses, pure membranes displayed much higher strength and lower flexibility compared to the blended membranes. However, interestingly, a comparison of the tensile strength of the blended membranes indicates that the membrane containing the highest percentage of CTA (80%) has the least tensile strength, i.e., 10.04 ± 0.03 MPa. This is due to the formation of a loosely packed structure and the presence of voids between polymer chains, which is consistent with the findings of SEM (Figure 5).

### 3.2. Gas Permeation Study

**Figure 6:**
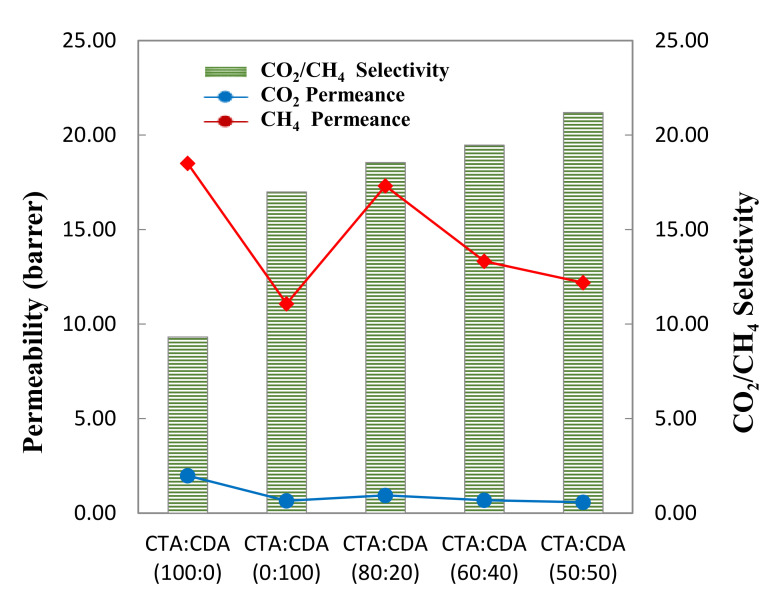
Separation performance of fabricated membranes as a function of blend composition.

As depicted in Figure 6, the pristine CTA showed better CO_2_ permeance and lower CO_2_/CH_4_ selectivity compared to pristine CDA at 5 bar. This might be because of the higher fractional free volume (FFV) of CTA (0.21) as compared to CDA (0.18) [28,29,30]. The replacement of hydroxyl groups with bulky acetyl groups opens up the polymer structure and reduces the inter-chain packing density, resulting in higher gas permeability [31]. Moreover, acetyl groups present in the polymer backbone chains have a loving affinity with CO_2_. The more acetyl groups there are, the greater the permeance of CO_2_. Since in the case of semicrystalline polymers, it is mainly gas transport that takes place through the amorphous region [32], so it was concluded that the intrinsic amorphous phase permeance of CTA is much higher than CDA that even the smaller amorphous region of CTA exhibited pronounced permeation performance.

Furthermore, it was observed that increasing the CDA content in CTA/CDA blend resulted in the improvement of CO_2_/CH_4_ selectivity coupled with a slight reduction in the permeability. The 80/20 (wt.%) CTA/CDA blend films had a CO_2_/CH_4_ selectivity of 18.55, which was 98% higher than the pristine CO_2_/CH_4_ selectivity of CTA. However, the CO_2_ permeability was reduced around 6%. Decreased permeance is attributed to the lower sorption rate of CO_2_ due to the lesser concentration of acetate groups. Moreover, steric hindrance that was created mainly by the acetate groups was also reduced, which leads to better packing density results in lower permeability and higher selectivity [13,33]. The findings are in accordance with the abovementioned analysis. The gas separation performances of all fabricated membranes are summarized in Table 3.

### 3.3. Performance Comparison of Fabricated Membranes

**Figure 7:**
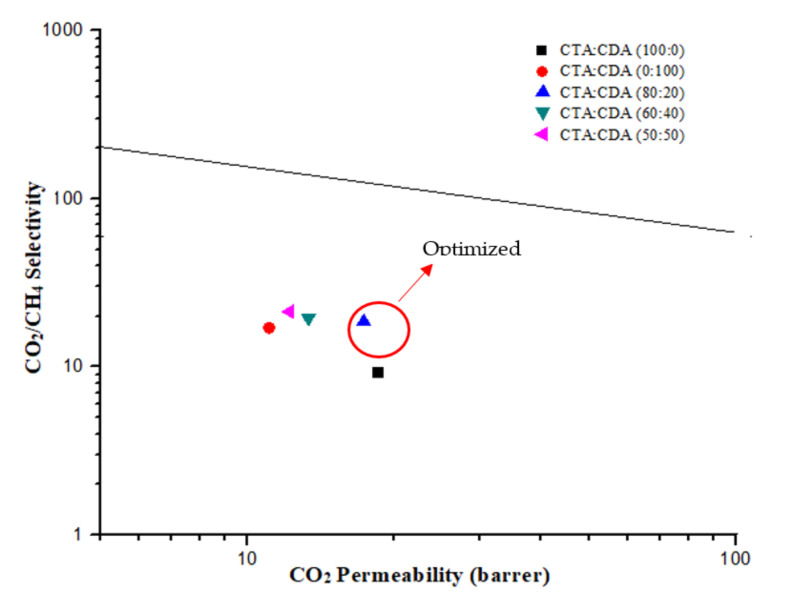
Performance comparison of fabricated membranes on Robeson’s upper bound (2008).

The separation performance of all fabricated membranes was compared based on 2008 Robeson’s upper bound curve as depicted in Figure 7. When the chains of CDA and CTA were linked together physically, various types of intermolecular molecular interactions occurred that affected the transport properties in a way where CTA:CDA (80:20) had acquired a better position in Robeson’s upper bound curve.

### 3.4. Plasticization Pressure

For the membranes made from glassy polymers, there are three different types of pressure dependencies of CO_2_ permeability [34]. Type A shows a decreasing trend of permeability versus pressure and does not exhibit any plasticization phenomenon. Type B exhibits plasticization at a certain value of pressure, and the permeability first decreases with pressure increase, up to a certain threshold value. Above the plasticization pressure, the gas permeability increases with feed pressure increment. Type C shows an increasing trend between the permeability and the feed pressure mainly owing to the rubbery characteristics of glassy polymers [34,35].

**Figure 8:**
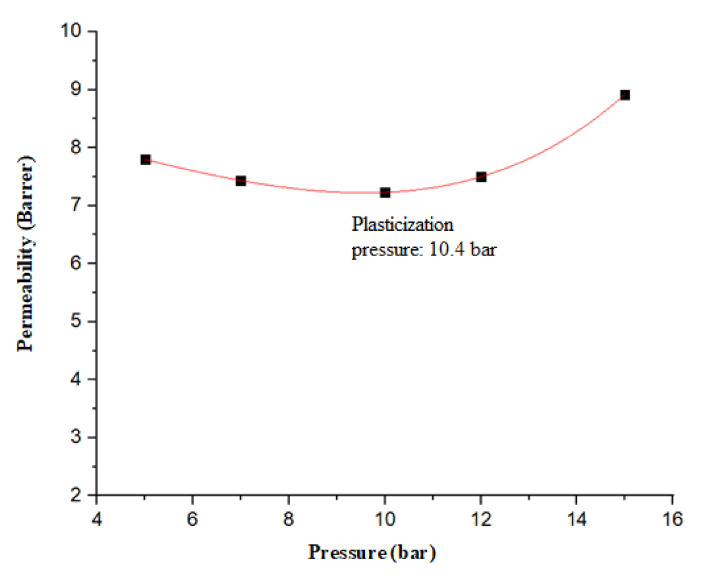
Effect of CO_2_ pressure on permeance.

In order to figure out the type of pressure dependency of CO_2_ permeance in the present work, the feed pressure was varied from 2 to 12 bar for optimal CTA:CDA (80:20). Referring to Figure 8, a Type B relationship was exhibited by the fabricated membrane. Initially, the CO_2_ permeance exhibited a small reduction up to the minimum value and then showed an increase. The reduction in the permeance with the increase of the feed pressure is well explained in literature by the dual sorption model [8,36]. However, when above a certain value of pressure, the CO_2_ causes the polymer to swell to such an extent that polymer chains become flexible and the free volume increase, consequently increasing the CO_2_ permeance [37]. The pressure corresponding to the minimum permeance is termed as “plasticization pressure”. The CO_2_ permeance data were fitted by the second order polynomial to figure out the curve trend. A plasticization pressure of 10.48 was recorded. The results are in good agreement with the literature [18].

## 4. Conclusions

Flat sheet membranes were prepared by blending cellulose acetates of a degree of acetylation, i.e., 2.45 and 2.84. Fabricated membranes were characterized by SEM, FTIR, UTM, and XRD. The formation of a homogenous blend, good interfacial interaction, dense and defect-free membranes were confirmed from the SEM results. All the CTA/CDA blended membranes were asymmetric with a loosely packed structure in between dense skin layers. FTIR results verified the presence of physical interaction between CTA and CDA polymeric chains. Tensile strength as well as the flexibility of membranes were greatly reduced by the formation of blend, as confirmed from the UTM results. The gas permeation study for CO_2_ and CH_4_ was carried out using a single gas at 5 bar. Significant improvement in the CO_2_/CH_4_ selectivity coupled with slight reduction in permeability was recorded by blending CDA in CTA. The separation performance of all the fabricated membranes was compared based on Robeson’s upper bound curve (2008). We found that CTA:CDA (80:20) had acquired a better position in Robeson’s upper bound curve; it exhibited a CO_2_ permeability of 17.32 barrer and a CO_2_/CH_4_ selectivity of 18.55. Comparing with the pristine CTA membrane, the optimal blended membrane showed 98% enhancement in CO_2_/CH_4_ selectivity with only 6% reduction in CO_2_ permeability. It was concluded from the current work that the fabricated membranes have great potential for the separation of CO_2_ and CH_4_.

## Figures and Tables

**Table 1:** Comparison of dense film properties of CDA and CTA.

Properties	CDA	CTA
Glass transition temp. (T_g_ °C)	187	185
Melting temp. (T_m_ °C)	233	293
Crystallinity (%)	37	52
Tensile strength (103 MPa)	12.7	14
Elongation at break (%)	14	17

**Table 2:** Comparison of mechanical performance.

Name of Sample	Tensile Strength (MPa)	Elongation (%)
CTA:CDA (100:0)	38.55 ± 0.22	5.56 ± 0.32
CTA:CDA (0:100)	32.89 ± 0.41	6.47 ± 0.56
CTA:CDA (80:20)	10.04 ± 0.03	10.06 ± 0.32
CTA:CDA (60:40)	11.98 ± 1.2	8.24 ± 0.99
CTA:CDA (50:50)	12.68 ± 0.82	7.50 ± 0.20

**Table 3:** Gas separation performance of fabricated membranes.

Sample Name	CO_2_ Permeability (Barrer)	CH_4_ Permeability (Barrer)	CO_2_/CH_4_ Selectivity
CTA/CA (100:0)	18.50 ± 0.12	1.98 ± 0.14	9.33
CTA/CA (0:100)	11.08 ± 0.42	0.65 ± 0.22	17.00
CTA/CA (80:20)	17.32 ± 0.09	0.93 ± 0.18	18.55
CTA/CA (60:40)	13.33 ± 0.52	0.68 ± 0.62	19.48
CTA/CA (50:50)	12.20 ± 0.25	0.58 ± 0.17	21.20
